# Late abdominal pregnancy in a post-conflict context: case of a mistaken acute abdomen - a case report

**DOI:** 10.1186/s12884-020-02939-3

**Published:** 2020-04-22

**Authors:** Justin Lussy Paluku, Benjamin Kambale Kalole, Cathy Mufungizi Furaha, Eugenie Mukekulu Kamabu, Gaspard Makambo Mohilo, Benjamin Kasereka Kataliko, Susan Andrea Bartels

**Affiliations:** 1 Department of Obstetrics and Gynecology, University of Goma, Goma, Democratic Republic of Congo; 2 Department of Obstetrics and Gynecology, HEAL Africa Hospital, Goma, Democratic Republic of Congo; 3 Departments of Emergency Medicine and Public Health Sciences, Queen’s University, Kingston, Canada

**Keywords:** Abdominal pregnancy, Painful fetal movements, Acute abdomen, Post-conflict

## Abstract

**Background:**

Abdominal pregnancies have been reported in both high-income countries as well as low- and middle-income countries. They are frequently missed in routine antenatal care in resource-limited settings and delayed diagnosis is usually associated with poor fetal and maternal outcomes including death. This case report is among the first from eastern Democratic Republic of Congo (DRC), a post-conflict region.

**Case presentation:**

In this case study, we present a 25 year-old primigravida patient referred to HEAL Africa hospital for management of an acute abdomen at 33-weeks gestation. Her chief complaint was severe abdominal pain associated with each fetal movement for a period of 1 week prior to admission. A diagnosis of peritonitis was made. Emergency laparotomy revealed a normal live 2 kg baby with placental implantation on the greater omentum and small intestine mesentery. The placenta was not removed. Both maternal and fetal outcomes were good.

**Conclusion:**

Abdominal pregnancy with a normal live fetus at such an advanced gestational age is rare. This case reminds clinicians that abdominal pregnancy remains a differential diagnosis for painful fetal movements.

## Background

Abdominal implantation of a pregnancy is uncommon, accounting for 1.4% of all ectopic pregnancies and with a reported range of 1:10000 to 1:30000 pregnancies [1–3]. Abdominal pregnancies have been reported in several contexts in both high-income countries as well as low- and middle-income countries. A review of 163 cases of abdominal pregnancies from 13 countries emphasized how difficult it is to make the diagnosis [4]. Another series of 19 cases from Libreville, Gabon also highlighted the challenges of timely accurate diagnosis beyond the second trimester, especially in resource limited settings [5].

Severe complications associated with ectopic pregnancies are well known including maternal and fetal death, especially with increased gestational age. With abdominal pregnancies, fetal mortality rates range from 40 to 95%, while maternal mortality ranges from 1 to 18% [2]. Because diagnosis is typically made late, the fetus is often already dead when the abdominal pregnancy is recognized [2, 5]. With increasing gestational age, maternal complications can occur at any time in the antepartum, peripartum or postpartum periods. These complications include spontaneous separation of the placenta leading to massive haemorrhage, shock, disseminated intravascular coagulation [3], organ failure, and death. Also, attempts to remove the placenta may cause uncontrollable, catastrophic bleeding leading to maternal death. After delivery, an in-situ placenta may not resorb, and infection may develop from its necrosis. However, good maternal and fetal outcomes have also been documented at a range of gestational ages from 31 weeks [6–8] to 39 weeks [3, 5].

Existing literature around abdominal pregnancies is mostly comprised of case reports and case series. However, this case report is among the rare ones from eastern Democratic Republic of Congo (DRC), a post-conflict region. Despite a positive outcome for both the mother and baby, neither the ultrasound nor the physical exam revealed the diagnosis of abdominal pregnancy pre-operatively. This case report alerts clinicians to be aware of the possibility of abdominal pregnancy in a patient with painful fetal movements and contributes to existing data about good fetal and maternal outcomes.

## Case presentation

A 25 year-old primigravida patient was referred from a nearby health center to HEAL Africa Hospital, a multidisciplinary tertiary hospital for management of an acute abdomen at 33-weeks gestation.

Her chief complaint was severe abdominal pain associated with each fetal movement for a period of 1 week prior to admission at the referring health center. Among other undocumented treatments, the patient had been managed with spasmolytics and hematinics but without relief of her pain. On August 30, 2019, when the patient’s condition worsened, the decision was made to transfer her for further care. She reported to the emergency unit of the hospital on the same date at 21h30.

Her first and second trimesters had been uncomplicated. She had received antenatal care in a nearby clinic where she was treated with antihelmintics, iron and folic acid supplementation, as well as prophylactic anti-malarials, according to recommended standards. The patient had not had an obstetrical ultrasound. There was no history of any symptoms suggestive of sexually transmitted diseases such as vaginal discharge or genital ulcers.

about the duration of her cycle, she typically bled for 3 days per cycle and denied pain or passage of clots during menstruation. Dates of her last normal menstrual period were unknown.

Past medical history was notable for malaria with two prior admissions to a nearby clinic. There was no history of chronic illnesses such as hypertension, diabetes mellitus, asthma or sickle cell disease, and the patient did not take any medications on an ongoing basis. She had never been tested for HIV.

She had never undergone myomectomy or any other surgical procedures and did not have a history of blood transfusion. There was no history of involvement in road traffic or other accidents.

On social history, the patient was the third born in a family of five children. Her parents and siblings were alive and healthy. There was no family history of chronic illnesses. The patient was a married housewife and did not smoke cigarettes or drink alcohol.

In summary, this was a healthy 25-year old primigravida who was admitted with a one-week history of severe abdominal pain associated with fetal movements at 33- weeks gestation in an otherwise uncomplicated pregnancy.

On physical examination, the patient was noted to be ill-appearing. Vitals signs were as follows: heart rate of 99 beats per minute, blood pressure of 120/69, respiratory rate was 22 breaths per minute, oxygen saturation of 98% on room air and temperature of 36.9^0^ C.

The patient’s pulse was regular and of normal volume. Apex beat was noted in the 5th intercostal space with normal S1 and S2 on auscultation. Chest expansion was symmetrical and breath sounds were normal with bilateral good air entry.

The abdomen was symmetrical but tense without surgical scars. Striae gravidarum and a linea nigra were visible. There was marked tenderness on abdominal palpation, particularly in the peri-umbilical area and associated with each fetal movement. Palpation of the liver, spleen, and kidneys was limited due to the patient’s tenderness. Fundal height was not well delineated but was estimated at 28/40 weeks. There were no palpable contractions but marked abdominal tenderness was noted during fetal movement. Fetal parts were not easily palpable through the abdominal wall. Additionally, fetal presentation and fetal lie were not easily appreciated on physical exam. A regular fetal heart of 148 beats per minute was auscultated in the mesogastrium.

Examination of the vulva and vagina were normal. The cervix was long, posterior, and not excitable. The os was closed. No abnormal discharge was noted.

Diagnosis of an acute abdomen in the third trimester of pregnancy was made and acute peritonitis was suspected. Differential diagnosis included appendicular or other bowel perforation.

The patient was admitted to hospital. An emergency ultrasound showed a single viable pregnancy at 33-weeks gestation with a low-lying placenta and oligoamnios. Initial hemoglobin was 8.1 g/dl with a hematocrit of 22.6%.

After intravenous access was obtained, intravenous fluids and initial pain management were started. The patient was counselled and consented for an emergency laparotomy.

**Fig. 1:**
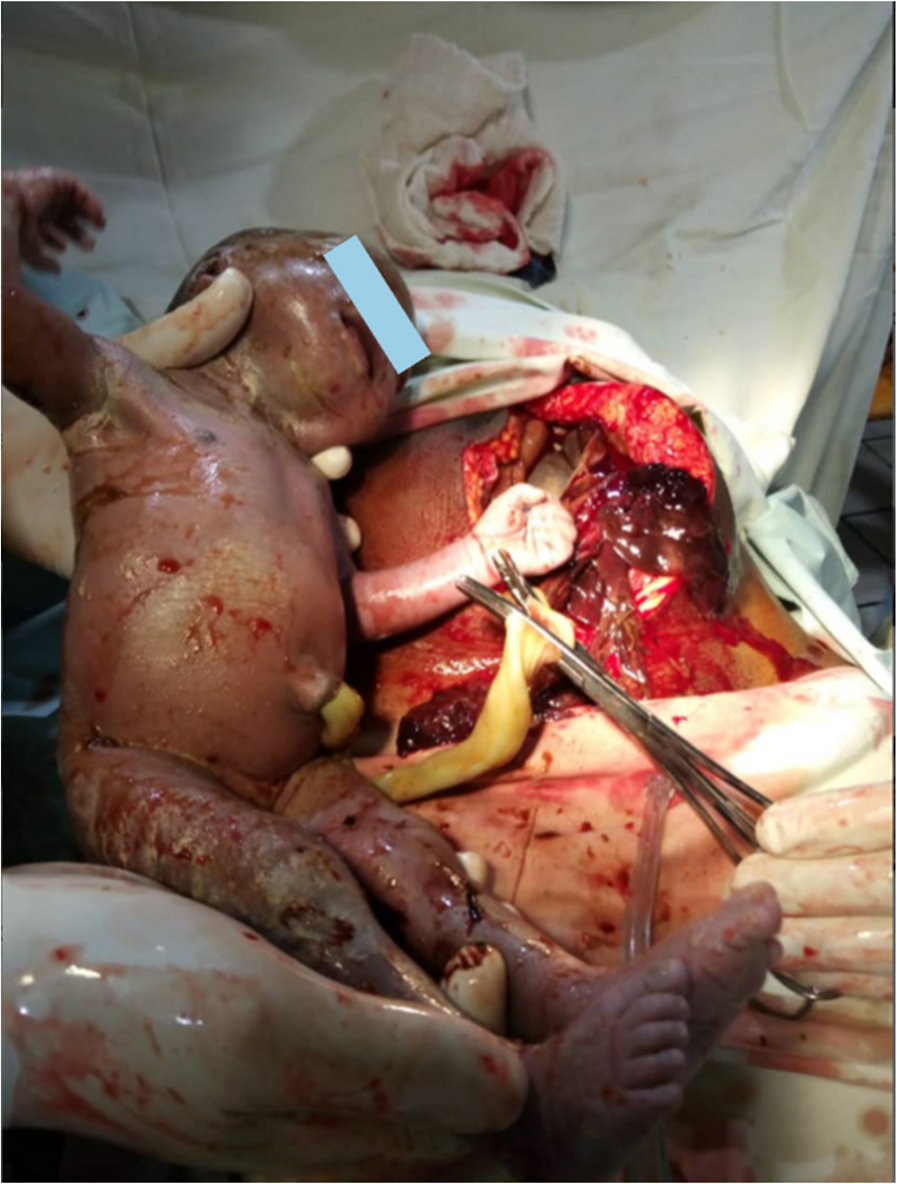
Baby being delivered from the abdominal cavity

**Fig. 2:**
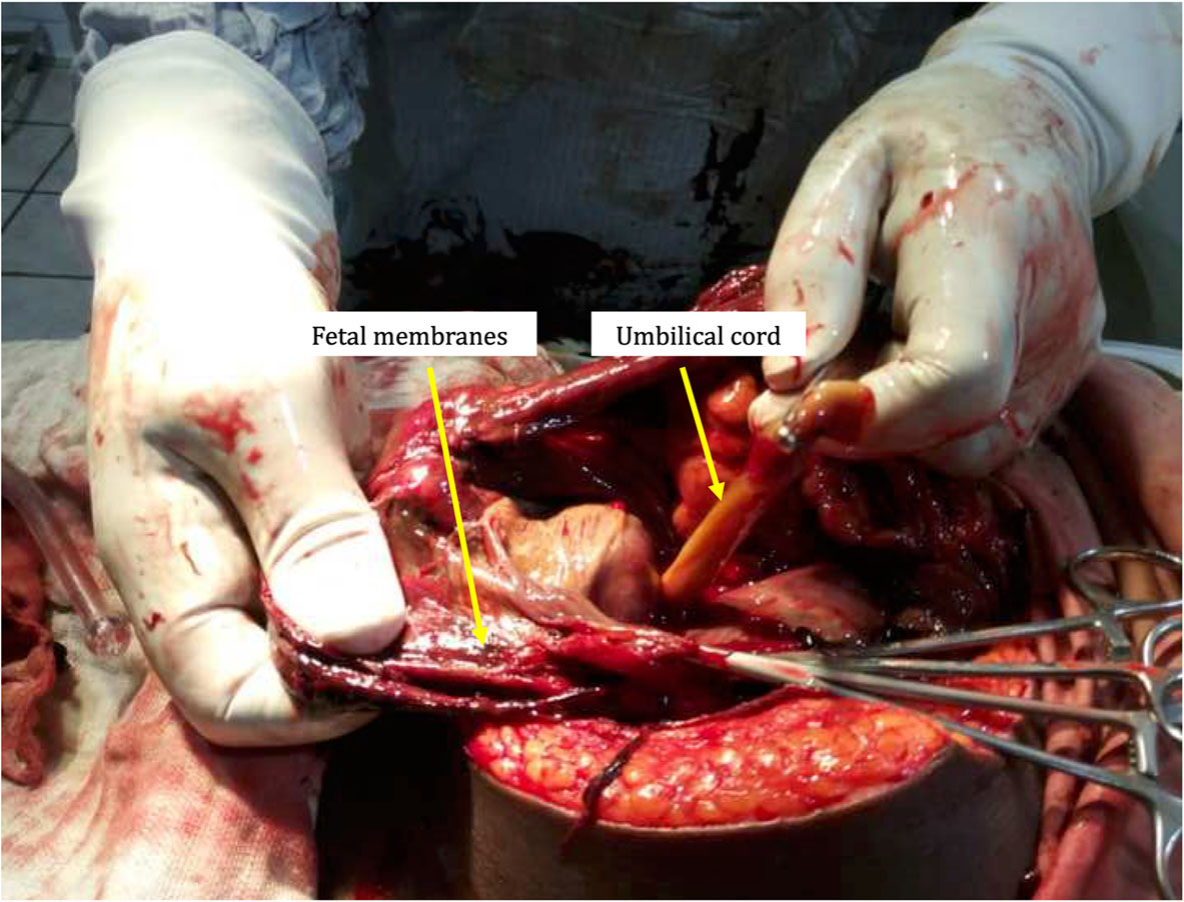
Placenta implanted on the omentum and small intestine mesentery

She was taken to operating theatre and was given general anesthesia with endotracheal intubation. Both an obstetrician and a general surgeon scrubbed for the case. A sub-umbilical incision was made and then extended above the umbilicus. On entering the abdominal cavity, a huge reddish mass was identified. Fetal parts were visible through the membranes delineating the mass. There was minimal meconium stained amniotic fluid around the baby. Upon digital opening of the mass, a live 2000 g female baby in longitudinal lie with the head in the maternal pelvis was delivered [Fig. 1]. APGAR scores were 8, 6, and 9 at 1, 5 and 10 min respectively. The baby was immediately taken to neonatology for thorough screening by the paediatrics team and was found to be healthy with no congenital abnormalities. Careful exploration of the abdomen revealed a placenta implanted on the greater omentum and on the small bowel mesentery [Fig. 2]. There was no plane of cleavage and any manoeuver to remove the placenta was susceptible to bleeding. It was decided to leave the placenta in place. Membranes were stripped and the umbilical cord was cut near its placental insertion. The patient remained hemodynamically stable throughout the surgery and no complications were noted. However, she did receive one unit of whole blood transfusion.

The patient was admitted to the post-partum ward after she was fully awake and was started on parenteral antibiotics and analgesics for 3 days, after which she was transitioned to oral treatment. Patient was also started on hematinics. Weekly ultrasounds were planned as well as serial quantitative beta-HCG measurements to evaluate the status of the placenta.

### Post-partum week 1

The patient remained stable in the post-operative period. Her first post-partum ultrasound showed an intra-abdominal placenta in the hypogastric region extending to the left and right iliac fossas with evidence of vascularisation on Doppler. Partial placental detachment was noted with two pouches of encapsulated peri-placental hematoma having maximal diameters of 9.39 cm and 6.78 cm each. The anteverted, anteflexed and empty uterus was well visualized and the serum beta – HCG was > 1500 IU/ml.

### Post-partum week 2

In the second post-operative week, the patient did not have any major complaints. Ultrasound again demonstrated an intra-abdominal placenta in the hypogastrium, above the uterus, with an encapsulated peri-placental hematoma of 8.41 cm in the longest diameter. The uterus remained empty, anteverted, anteflexed. Serum beta–HCG returned at 653.9 IU/ml.

### Post-partum week 3

The patient continued to make a good recovery and the baby appeared to be healthy. The mother was discharged home on prophylactic antibiotics for 1 week with a planned follow up at the end of post-partum week 6.

## Discussion and conclusion

Abdominal pregnancy is categorized as either ‘early’ (≤ 20-weeks gestation) or ‘late’ (> 20-weeks gestation) depending on the gestational age at which the diagnosis is made. Abdominal pregnancy is often fatal to both the fetus and the mother [1, 9] and careful management is required in order to save their lives [10]. Although proper diagnosis is needed to improve outcomes, unfortunately the diagnosis of abdominal pregnancy is often missed in routine antenatal care [7]. This is partially due to nonspecific symptoms such as abdominal pain, nausea, vomiting, easily palpable fetal parts, fetal malpresentation, pain on fetal movement, and displacement of the cervix [11]. A previous case report of abdominal pregnancy in the DRC has been published. Like in many other reported cases, the diagnosis was made late (after 36 weeks gestation) and the fetus was already dead [12].

Our patient presented with severe abdominal pain during fetal movements. The fetal presentation was not well defined on exam. Although easy palpation of fetal parts has been reported as a sign of abdominal pregnancy, this was not possible in the current case because the patient had a tense abdomen. Complaint of painful fetal movements noted in this case is consistent with findings noted in earlier case series. For instance, in a report by Bohiltea et al. painful fetal movements were noted in 40% of abdominal pregnancies [13].

Making the diagnosis of abdominal pregnancy is challenging. While in more developed settings, magnetic resonance imaging (MRI) is the diagnostic method of choice for abdominal pregnancy, particularly in the more advanced stages [2, 14], in low- and middle-income countries, clinicians must rely on good clinical judgement, and where available, on ultrasound [1, 10]. When performed in the first trimester, ultrasound will show an empty uterus with a separate gestational sac, or with a mass separated from the uterus, adnexa, and ovaries. Suspicion of abdominal pregnancy is increased by the presence of symptoms like abdominal pain with a positive pregnancy test. In the second and third trimesters, ultrasound usually shows no uterine wall surrounding the fetus, fetal parts that are very close to the abdominal wall, abnormal lie and/or no amniotic fluid between the placenta and the fetus [3]. Unfortunately, ultrasound may not be as helpful at more advanced gestational ages [10] like in the current case, where neither clinical assessment nor ultrasound made the diagnosis. It is also noted that transvaginal ultrasound in the first trimester of pregnancy may improve the diagnosis of extrauterine pregnancy. Importantly, in patients with previous major uterine surgery, differential diagnosis of abdominal pregnancy must include a uterine rupture with extrusion of the products of conception into the peritoneal cavity [15]. Our patient had not had an ultrasound in either the first or second trimesters. We therefore propose that in resource-limited settings, where ultrasound is not always available, painful fetal movements may be an important sign of abdominal pregnancy and should raise the index of suspicion for health care providers.

Abdominal pregnancies may progress uneventfully to an advanced stage, particularly in resource-limited countries [9] but they rarely reach term [3, 10]. Possible factors believed to contribute to fetal survival include the site of implantation and availability of adequate vascular supply [3]. We presume that restricted blood supply and oxygenation results in poor weight gain and fetal demise before birth. Furthermore, congenital malformations, which appear to be more common in abdominal pregnancies, also contribute to fetal demise [6, 9–11]. In our case, the pregnancy progressed to 33 weeks, likely because implantation occurred on the well-vascularized omentum and small bowel mesentery, resulting in an adequate birth weight of 2 kgs at 33 weeks. In recent years, a few case reports have described abdominal pregnancies with implantation on the liver [16], spleen [17] and kidney [18] but these abdominal pregnancies were diagnosed and terminated in the first trimester. However, several other existing case reports describe delivery of a normal live fetus in different African countries such as Ghana [10], Ethiopia [1], and Nigeria [6, 9].

The diagnosis of abdominal pregnancy may be a surprise finding in the operating theatre, as was the case with our patient. For instance, some abdominal pregnancies are diagnosed at the time of emergency caesarean section for failed labor induction [2] and during elective caesarean section [7]. This is more common in low resource settings where access to imaging such MRI, and in some contexts ultrasound, is not possible. Other abdominal pregnancies are diagnosed during exploratory laparotomy [4] as occurred in the current case.

Abdominal pregnancy is associated with high maternal and fetal morbidity and mortality. Early diagnosis and timely intervention are crucial. Consensus on the best management of abdominal pregnancy is lacking. A conservative approach with delayed surgery is suggested when patients present after a gestational age of 24 weeks with a live fetus [7, 19]. In these cases, timing of delivery should be decided in consultation with the mother once the fetus has reached a viable age, because perinatal death may result from either prematurity or prolonged gestation in a compromised environment [4].

Management of the placenta is crucial. The decision to remove or to leave it in situ depends on the intraoperative findings. Most authors agree that the placenta should be removed provided its blood supply is identified and can be ligated without damaging other organs. Otherwise attempts to remove an abnormally implanted placenta may result in catastrophic hemorrhage that can lead to maternal death. Also measures taken to control intra-operative hemorrhage may compromise the blood supply of other organs. In these cases, it is recommended to leave the placenta in situ, where it may resorb spontaneously. If it does not, however, there is a risk of complications such as infection or necrosis, sometimes requiring a second surgery [4, 19]. In our case, the placenta was left in situ as there was no clear plane of cleavage that would have allowed its safe removal, and because the blood supply could not be well identified for safe ligation.

With the placenta left in situ, our patient was discharged home on prophylactic antibiotics. It is crucial to follow up patients with undelivered placentas until the beta hCG returns to zero, due to the possibility of complications such as infection. It can take some time for the beta hCG concentration to become undetectable, and in one expectantly managed placenta increta case, it took 22 weeks [20]. With the use of serial ultrasonography in the postpartum period, continuous decreases in the size of the placenta is also reassuring for placental resorption. Two ultrasound examinations were done in our patient at 1 and 2 weeks postpartum, and the placenta was found to be decreasing in size. Additionally, weekly beta hCG testing showed a decreasing concentration.

In summary, diagnosing abdominal pregnancy remains a challenge, particularly in low-resource settings where ultrasound and/or MRI is less accessible. This case reminds clinicians that abdominal pregnancy remains a differential diagnosis for painful fetal movements.

## Data Availability

Anonymized data are available from the corresponding author on reasonable request.

## Abbreviation

MRI: Magnetic Resonance Imaging
